# Post-traumatic Stress Disorder Symptoms Resulting from Torture and Other Traumatic Events among Syrian Kurdish Refugees in Kurdistan Region, Iraq

**DOI:** 10.3389/fpsyg.2017.00241

**Published:** 2017-02-20

**Authors:** Hawkar Ibrahim, Chiya Q. Hassan

**Affiliations:** ^1^Department of Psychology, Clinical Psychology and Psychotherapy, Bielefeld UniversityBielefeld, Germany; ^2^Department of Clinical Psychology, Koya UniversityKoya, Iraq

**Keywords:** PTSD, torture, Kurd, conflict, refugees

## Abstract

Political violence is known to cause psychological distress. There is a large body of empirical studies drawing correlations between war trauma, torture, and post-traumatic stress disorder (PTSD). However, there are few studies on the effects of war-related trauma among Syrian refugees after events following the ‘Arab Spring’ uprisings between 2010 and 2012. This study examines the association of PTSD symptoms with torture and other traumatic events among Syrian Kurdish refugees living in Kurdistan Region, Iraq. The experiences and PTSD symptoms among 91 Syrian Kurdish refugees in the Arbat camp in the Sulaymaniyah Governorate of the Kurdistan Region of Iraq were assessed using the Harvard Trauma Questionnaire, sections I, IV, and V. Results showed that the estimated levels of PTSD symptoms were high: between 35 and 38%. There were no significant gender differences in the occurrence of PTSD symptoms. However, men reported more general traumatic experiences than women. There were significant positive correlations between PTSD symptoms with traumatic events and torture (*r* = 0.500, *r* = 0.366, respectively). Examining the mental health impact of torture and other traumatic events among refugees has possible implications for organizations managing rehabilitation programs for individuals who have been exposed to traumatic events.

## Introduction

Over the past 5 years, the population of Syria has faced numerous civil wars and political conflicts. Thousands of people have died, and millions have fled across the border to Lebanon, Turkey, Jordan, and Iraq, including the semi-autonomous Kurdistan Region of Iraq (KRI). According to official statistics from the United Nations High Commissioner for Refugees (UNHCR) on 31 December 2014, more than 225,000 Syrian refugees have sought refuge in the KRI since the war began. These refugees are dispersed into nine camps in the cities of Erbil, Sulaymaniyah and Duhok (UNHCR and REACH Initiative, 2015). Salman’s (2012) assessment of the situation of the Syrian refugees in the KRI found that most of those he interviewed had selected the fled to the KRI instead of to other countries for two main reasons; first the “safety and stability enjoyed by the province,” and second for “the fact that people of the province of the same nationality and religion and using the same language” (p. 15).

It is well-known that war in itself can lead to a range of other traumatic experiences, such as witnessing extreme violence, terrorist attacks, kidnappings, torture, separation from one’s family and forced migration (Johnson and Thompson, 2008). Studies indicate that most civilian adults and children in war-affected zones, including those in the Middle East, experience at least one traumatic event as a result of war and political conflict (Qouta and El Sarraj, 2004; Khamis, 2005; Fasfous et al., 2013).

Negative mental health consequences of war-related traumatic events are well-documented within current psychological literature. Most studies in post-conflict settings and among war-affected populations indicate a positive association between war trauma and the presence of various mental health disorders. For example, Priebe et al. (2010) examined mental disorders following the war in five countries (Bosnia-Herzegovina, Croatia, Kosovo, the Republic of Macedonia, and Serbia) and found that potentially traumatic experiences during and after the war were associated with higher rates of mood and anxiety disorders. Al-ghzawi et al. (2014) reviewed nine studies related to the impact of war and conflict on mental health among populations in Arab countries. They found a significant impact of war trauma on mental health. In addition, they found that post-traumatic stress disorder (PTSD) was one of the most common psychological complications among war trauma victims. Ayazi et al. (2014) examined the association between exposure to traumatic events and anxiety disorders in the post-conflict setting in South Sudan. They reported that exposure to trauma was significantly associated with diagnoses of anxiety. Similar association was found by Farhood et al. (2013) when they explored the impact of war-related life events on well-being among civilian population in southern Lebanon. More recently, Atwoli et al. (2015) reviewed epidemiological surveys of traumatic events and found high PTSD prevalence rates in post-conflict settings.

In addition to war-related traumatic events, refugees may also experience different types of torture (Gorst-Unsworth and Goldenberg, 1998; Masmas et al., 2008; Heeren et al., 2012; Widmann et al., 2014). In reviewing 40 years of health science research, Green et al. (2010) found that definitions by the World Medical Association and the United Nations were most often utilized in the scientific literature for torture. According to the World Medical Association’s Tokyo Declaration, torture is defined as “the deliberate, systematic or wanton infliction of physical or mental suffering by one or more persons acting alone or on the orders of any authority, to force another person to yield information, to make a confession, or for any other reason” (World Medical Association, 1975, p. 1). The United Nations defined torture in article 1.1 of the Convention Against Torture as “any act by which severe pain or suffering, whether physical or mental, is intentionally inflicted on a person for such purposes as obtaining from him or a third person, information or a confession …” (Wendland, 2002, p. 23). Many scholars (e.g., Kastrup et al., 1986; Elsass, 1997; Gorman, 2001) have investigated the purposes of torture. They note that the primary aim of torture is not only to obtain information from the victims but also to break down the identities and personalities of those tortured.

Empirical psychological studies in the field of trauma have shown several mental health sequelae of torture. The most frequent psychological disorders among torture survivors were PTSD, generalized anxiety disorder, depression, and somatic disorders (Shrestha et al., 1998; Van Ommeren et al., 2002; Elklit et al., 2012; Tufan et al., 2013). Other psychosocial problems were also mentioned, such as insomnia, isolation, and loneliness (Bolton et al., 2013).

Due to exposure to war trauma, torture and post-migration living difficulties, refugees are more likely to develop mental health disorders (Keller et al., 2006; Schweitzer et al., 2006; Onyut et al., 2009; Badri et al., 2012; Bogic et al., 2012; Aragona et al., 2013).

Alpak et al. (2015) studied trauma and PTSD among Syrian adult refugees in Turkey aged between 18 and 65 years. They found that participants experienced between 0 and 9 traumatic events and PTSD was present in 33.5% of their sample. Naja et al. (2016) interviewed Syrian refugees and found that the prevalence of current depression was 43.9%. More recently, Kazour et al. (2016) examined PTSD among Syrian adult refugees in Lebanon and found 35.4% of lifetime prevalence and 27.2% of point prevalence of PTSD.

Epidemiological studies suggest gender differences in the prevalence of trauma and PTSD. Research suggests that males are more likely to experience traumatic events and that females are more likely to develop PTSD (Keane and Kaloupek, 2002; Kimerling et al., 2007; Wittchen et al., 2009). However, ethnocultural issues may also play a significant role in gender differences. Some studies from Middle Eastern countries have found no gender differences in PTSD (Elbedour et al., 2007; Shaar, 2013). The differences between findings in Middle Eastern and studies carried out in other regions require further attention.

Political violence is known to cause psychological distress, and there is a large body of empirical studies drawing a significant association between war trauma, torture, and PTSD. On the other hand, studies also showed that the traumatized people report positive psychological changes in the aftermath of trauma in their social and personal levels such as well-being, psychological growth, sense of coherence, and adaptive adjustment (Veronese and Pepe, 2015; Sleijpen et al., 2016).

However, cross-cultural studies showed negative impacts of war-related trauma but to date there is limited data on the mental health among Syrian refugees and this represents a serious gap in our knowledge. Our study is part of a larger investigation aimed to see if the expected pattern of war-trauma and the dose-effect model could also be confirmed in this population.

The current study aimed to examine the levels of PTSD symptoms among Kurdish Syrian adult refugees and the relations between torture and traumatic experiences with PTSD symptoms using a quantitative approach. We hypothesize that Kurdish refugees fleeing Syria will experience multiple types of war-related trauma and torture. Furthermore, the study aims to examine gender differences in PTSD symptoms, torture and the experience of traumatic events. We hypothesize that there will be gender differences in frequency of torture, traumatic events, and PTSD.

## Materials and Methods

### Participants

Participants in the research were Syrian Kurdish refugees living in Arbat Camp in Sulaymaniyah Governorate in the KRI. The refugees in this camp originally come from predominantly Kurdish regions of Syria, and fled to the KRI as result of civil war, terrorist attacks, and air strikes. The inclusion criteria for the study were: (1) aged 18 or older; (2) registered as a refugee by UNHCR at least 3 months before the interview. None of the refugees refused to participate in the study. The participants consisted of 100 Syrian Kurdish refugees. Nine participants were excluded from analyses because of missing data. Participants were aged between 18 and 57 years old (*M* = 29.91, *SD* = 9.54). Male participants constituted most of the sample (55%). The majority of the participants (60.4%) were married and 63.7% were unemployed at the time the research was conducted. In terms of education, only 5.5% of participants reported having no formal education. In terms of financial help and mental health services, 80.2% of participants received financial help but only 14.3% received formal mental health services (**Table 1**).

**Table 1:** Characteristics of participants.

Demographics		*N*	%
Gender	Female	40	44.0
	Male	51	56.0
Marital status	Never married	34	37.4
	Currently married	55	60.4
	Divorced	1	1.1
	Separated	1	1.1
Employment status	Employed	33	36.3
	Unemployed	58	63.7
Financial help	Received	73	80.2
	Not received	18	19.8
Mental health services	Received	13	14.3
	Not received	78	85.7
Missing family member or relatives	Yes	71	78.0
	No	20	22.0
Number of family members or relatives missing	Range: 0–25 people. *M* = 5.86, *SD* = 6.14		
Age	Range: 18–57 years old. *M* = 29.91, *SD* = 9.54		
Years of formal education	Range: 0^∗^–19 years. *M* = 8.86, *SD* = 3.95		
Number of children	Range: 0–13. *M* = 2.35, *SD* = 2.57		

*^∗^Illiterate and unschooled.*

### Procedure

Participants were recruited through community leaders in Arbat Camp. The interviews were conducted between January and March, 2014 in their own tents. Due to cultural considerations, verbal rather than written informed consent was obtained. In the local context, signing documents is associated with the bureaucracy of authoritarian states which could have raised suspicions among participants that information may be used for purposes other than scientific research. Moreover, some participants were illiterate. Verbal informed consent was obtained by using a standardized form which included information about voluntary participation, the right to withdraw without negative consequence, confidentiality and ensuring anonymity, reviewing the risks, benefits, and associated information of the study. The verbal consent of each participant was documented by the interviewers. This procedure and the protocols of this research were approved by the Ethical Committee of Koya University. After providing verbal consent, participants were asked to complete a background questionnaire followed by sections I, IV, and V of the Harvard Trauma Questionnaire (HTQ). For illiterate participants, questionnaires were read to them item by item and verbal answers were recorded.

### Measures

*The Demographic Data Questionnaire* consisted of two parts. The first part covered basic demographic variables (e.g., gender, age, marital status, etc.). The second consisted of some specific questions intended to solicit basic information related to refugees and their personal circumstances, such as the number of family members left behind, the date of leaving Syria, and whether they were in receipt of financial help or mental health services.

*The HTQ* is a self-report checklist designed by the Harvard Program for Refugee Trauma (HPRT) that investigates traumatic events, PTSD symptoms, and torture. There are numerous versions of this questionnaire but the present study used an Arabic version of this questionnaire (sections I, IV, and V) adapted for use by Shoeb et al. (2007) rather than a version using the Kurdish language. The Arabic version of the HTQ was chosen for two reasons; firstly, Arabic is the primary formal language in Syria; and secondly, before the civil war, governmental rules in Syria prohibited Kurds from learning Kurdish or building Kurdish language schools (Human Rights Watch, 1996). As a result, the majority of Syrian Kurds cannot read or write fluently in the Kurdish language.

The first section of the HTQ assesses trauma history before, during and after migration through 42 potentially traumatic events, answered in a “Yes/No” format. The fourth section consists of 45 items describing PTSD symptoms, using a four-point Likert scale rated from 1 (not at all) to 4 (extremely). This section is designed to evaluate different trauma symptoms that people may experience subsequent to hurtful or terrifying events in their lives. The first 16 items were derived from the DSM-IV criteria for PTSD and comprised of three subscales for three separate symptom clusters of PTSD; four items relate to re-experiencing symptoms; five items relate to symptoms of arousal and seven items to symptoms of avoidance. Symptoms scale scores are calculated as the mean score of the items (with a theoretical range of 1–4). The 29 additional symptom items were derived from clinical studies and experience. Generally, this part of the HTQ requests the responder to read each item carefully and decide how much the symptoms bothered them during the past week. The cut-off scores for PTSD diagnosis were greater than 2.5 (a mean score of >2.5). The final section of HTQ named “torture history” consists of 35 potential torture events with “Yes” or “No” answers. The internal consistency of HTQ subscales was shown to be high; the trauma symptoms subscale scored (Cronbach’s α ≥ 0.88) on 16 PTSD items and 0.94 on 45 items, traumatic events (Cronbach’s α = 0.90) and torture history section (Cronbach’s α = 0.89).

### Data Analysis

All statistical analyses were carried out by using the Statistical Package for the Social Sciences (SPSS) program version 20 for Microsoft Windows. Descriptive statistics (frequencies, means, and standard deviations) were used for analyzing all demographic variables, traumatic events, and PTSD symptoms. The differences between groups were analyzed with the two-tailed *t*-test. The correlations between the continuous variables were tested with Bivariate-Pearson correlation coefficients. The internal consistency reliability was determined using Cronbach’s Alpha. Normality of data was checked using Shapiro–Wilk’s test, Kolmogorov–Smirnov’s test with histograms and normal Q–Q plots (Ghasemi and Zahediasl, 2012). For adjusting *p*-value, we used Bonferroni correction (Bland and Altman, 1995). For overall tests (0.003) was set as a significance level for statistical analysis.

## Results

### PTSD Symptoms

Using the total 45 symptoms items, 35 participants had a mean score greater than 2.5 on HTQ (section V) indicating clinically significant symptomatology. On the first 16 PTSD item subscale of the HTQ, 32 of the participants had a mean score above the cutoff score of 2.5. Thus, the levels of symptoms of PTSD among participants according to established clinical cutoff scores on the HTQ (Arabic version) was between 35.16% (16 items) and 38.46% (45 items) (**Table 2**).

**Table 2:** Post-traumatic stress disorder (PTSD) symptomatology.

Measures	Cutoff score	*M*	Positive cases	Negative cases
PTSD 16	2.5	2.30	35.16%	64.84%
PTSD 45	2.5	2.25	38.46%	61.54%

### Torture Experience and Other Traumatic Events

**FIGURE 1:**
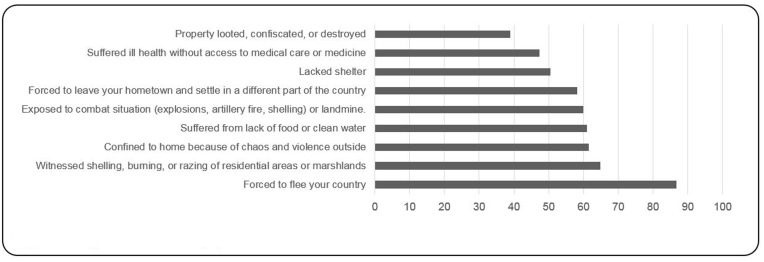
**Most frequent traumatic events (%)**.

**FIGURE 2:**
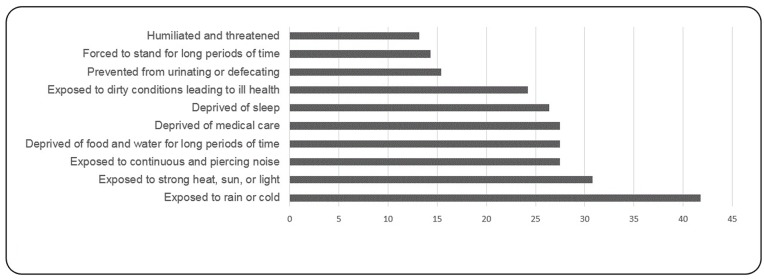
**Most frequent torture experiences (%)**.

Participants reported having experienced between 0 and 29 traumatic events (*M* = 11.12, *SD* = 7.37). Seventy-nine out of the participants reported having experienced at least three traumatic events listed in the first section of the HTQ during or after their migration. The most frequent traumatic events were “forced to flee your country,” reported by 79 participants (86.8%), “witnessed shelling, burning, or razing of residential areas or marshlands,” reported by 59 participants (64.8%), “confined to home because of chaos and violence outside,” reported by 56 participants (61.5%) (**Figure 1**). Regarding experiences of torture, participants reported having been exposed to between 0 and 24 torture events (*M* = 4.23, *SD* = 5.21). Thirty-eight out of the 91 participants reported having experienced at least two events of torture; 38 (41.8%) of participants were exposed to rain or cold, 28 (30.8%) exposed to strong heat, sun, or light and 25 (27.5%) were deprived of food and water (**Figure 2**).

The correlation between PTSD 16 items and 29 items with total 45 items of the HTQ section V were highly significant (*r* = 0.72, *p* = 0.000; *r* = 0.87, *p* = 0.000; respectively). As shown in **Table 3**, bivariate correlations showed statistically significant positive correlations between PTSD symptoms, traumatic events and torture (*r* = 0.500, *p* = 0.000; *r* = 0.366, *p* = 0.000; respectively). Similar significant positive relationships were found between each of the three PTSD symptoms clusters (re-experiencing, avoidance and hyperarousal) with traumatic events.

**Table 3:** Bivariate correlations between PTSD symptoms, traumatic events, and torture.

	1	2	3	4	5	6
(1) PTSD	—	0.827^∗∗^	0.904^∗∗^	0.917^∗∗^	0.500^∗∗^	0.366^∗∗^
(2) PTSD-RE		—	0.673^∗∗^	0.624^∗∗^	0.452^∗∗^	0.284^∗^
(3) PTSD-AR			—	0.729^∗∗^	0.360^∗∗^	0.274
(4) PTSD-AV				—	0.509^∗∗^	0.389^∗∗^
(5) Traumatic events					—	0.240
(6) Torture						—

*PTSD, PTSD- first sixteen items in section IV of HTQ; PTSD-RE, PTSD-re-experiencing symptoms; PTSD-AR, PTSD-arousal symptoms; PTSD-AV, PTSD-avoidance symptoms.*

*^∗^p = 0.003; ^∗∗^p < 0.001.*

Regarding gender differences in experiencing traumatic events, results showed that male participants had experienced more general traumatic events compared to females (*M* = 12.86 vs. 8.90 events). There were no statistically significant differences found between the genders in experiencing torture (females: *M* = 4.05, *SD* = 4.55; male: *M* = 4.37, *SD* = 5.71) two-tailed *t*-test (equal variances): *t*(89) = -0.292, *p* = 0.771. Similarly, no statistically significant correlation was found between age, traumatic events, and torture (*r* = -0.094, *r* = -0.039, *p* > 0.003).

### PTSD Symptoms and Demographics Variables

An exploratory data analysis was conducted to determine if the PTSD scores were normally distributed using the Explore procedure in SPSS Descriptive Statistics. Results from the Shapiro–Wilk’s test and Kolmogorov–Smirnov’s test along with a visual inspection of their histograms and normal Q–Q plots (Ghasemi and Zahediasl, 2012) showed that the PTSD scores were approximately normally distributed for all variables.

Regarding gender differences in PTSD symptoms, the results from two-tailed *t*-tests (unequal variances) showed that there were no significant differences in PTSD symptoms between females and males (Females: *M* = 2.25, *SD* = 0.589; Males: *M* = 2.34, *SD* = 0.818), *t*(88.420) = -0.620, *p* = 0.537. In addition, results showed no statistically significant differences of employment status on PTSD symptoms (employed: *M* = 2.39, *SD* = 0.145; unemployed: *M* = 2.24, *SD* = 0.654) two-tailed *t*-test (equal variances): *t*(89) = 0.941, *p* = 0.349. Similar results were reported in terms of receiving financial help and mental health services. Results from two-tailed *t*-test showed that there were no significant differences in PTSD symptoms between those refugees who received financial help and mental health services with those who had not received support: [Financial help: *M* = 2.26, *SD* = 0.685; non-financial help: *M* = 2.46, *SD* = 0.869) two-tailed *t*-test (equal variances): *t*(89) = -1.085, *p* = 0.281]; [mental health services: *M* = 2.35, *SD* = 0.7440; non- mental health services: *M* = 2.29, *SD* = 0.725) two-tailed *t*-test (equal variances): *t*(89) = 0.283, *p* = 0.778].

Additionally, there was no statistically significant correlation between PTSD symptoms according to age, education, number of children, and number of absent family members (respectively *r* = 0.004, *r* = -0.126, *r* = 0.089, *r* = -0.068, *p* = 0.972, *p* = 0.234, *p* = 0.403, *p* = 0.522).

## Discussion

This study examined the prevalence of PTSD symptoms among Syrian Kurdish refugees in association with torture and other traumatic events. Of the total sample, 38.46% reported PTSD symptoms in the clinical range using the 45-item total scale, and 35.16% met criteria on the first 16 symptom items of HTQ using established clinical cut-off scores. This finding is similar to results from a previous study by Alpak et al. (2015) reporting a frequency of PTSD of 33.5% among Syrian refugees in Turkey. It’s also in line with finding from meta-analyses, which have documented high levels of PTSD among refugees (Fazel et al., 2005; Bogic et al., 2015).

The results of the present study showed that both the number of traumatic events and instances of torture experienced was positively correlated with PTSD, supporting previous studies that showed similar correlations between traumatic events and PTSD symptoms (Silove et al., 1997; Karunakara et al., 2004; Badri et al., 2012) and between torture and PTSD (Van Ommeren et al., 2001, 2002; Piwowarczyk, 2007; Masmas et al., 2008).

A considerable number of studies have reported that PTSD is more common in women than in men; however, this was not supported by the current study, which found no statistically significant differences in PTSD by gender. Ethnocultural factors may play a significant role in the differences between our results and results from other studies. Most of these studies reported gender differences in PTSD were conducted in Western societies or by Western psychologists/psychiatrists among Kurdish and Arabic population using Western instruments for evaluating trauma and PTSD symptoms without validation. To determine what considered as a traumatic event requires cultural knowledge and awareness because culture plays a significant role in the way people perceive an event as traumatic (von Peter, 2008; Herbert and Forman, 2010). Interestingly, a recent meta-analysis of 10 studies of adolescents in Lebanon in times of civil war found no gender differences in PTSD (Shaar, 2013).

In addition, no gender differences were reported in the number of instances of torture experienced. Tolin and Foa (2006) quantitatively reviewed 25 years of research on sex differences in trauma. They revealed that males report higher levels of exposure to potentially traumatic events. This was supported by the present study showing that the male participants experienced more traumatic events as compared to female participants.

Only 14.3% of participants received mental health services and no significant differences were found between those refugees who received mental health services with those who did not, and this may potentially be attributed to the following two reasons. Firstly, the number of participants who have received mental health services is very small in comparison to those who have not received any mental health services. Secondly, it may potentially be related to the quality of services; because there is a serious lack of psychological services within the KRI. At the current time, there is only one graduate-level clinical psychology program in the whole region.

Several limitations exist in this study. The number of participants was small and only those refugees living in Arbat Camp in Sulaymaniyah Governorate, one of the smaller camps for Syrian refugees in the KRI, were examined. The findings of this study may have also been limited by the instruments used in this study, given that the participants were Syrian Kurdish refugees but the instruments used in this study were presented in the Arabic language. Moreover, there is also the possibility of bias by the participants in responding to the self-reported questionnaires because some of them may have believed that their answers would impact upon the possibility of receiving financial assistance. It was clearly explained to participants that no financial assistance would be provided as a result of their answers, but the possibility exists that some of the results may reflect this bias.

Further research into PTSD among Syrian refugees in the KRI, conducted with a larger sample size, may provide more informative findings. It will be more useful if future studies explore the association between war related events, torture and positive changes after trauma. Additionally, translation of existing trauma questionnaires into the Kurdish language may facilitate interviews and ensure accurate outcomes. Finally, our findings that between 35 and 38% of Syrian refugees are experiencing PTSD symptoms, lend weight to calls for improved mental health services for Syrian Kurdish refugees in the KRI.

## Conclusion

The results of this study supported findings from the literature about the positive significant association between PTSD symptoms, torture, and other war-related trauma. Our results did not show significant gender differences in the experience of PTSD symptoms. The findings of our study have possible applications for local and international governments, human right and mental health organizations, especially for those who provide psychosocial support programs for Syrian refugees. In addition, the results of our sturdy provide a better understanding of the mental health of Syrian refuges and this will contribute to the cross-cultural understanding of trauma and provide data to expand current models of trauma psychology. Also, such scientific documentation may contribute to increasing awareness of mental health needs and may provide an impetus for supporting expansion of psychological services in KRI and for Syrian refugees.

## Ethics Statement

This procedure and the protocols of this study were approved by the Ethical Committee of Koya University. Due to cultural considerations, verbal rather than written informed consent was obtained by using a standardized form which included information about voluntary participation, the right to withdraw without negative consequence, confidentiality and ensuring anonymity, reviewing the risks, benefits, and associated information of the study.

## Author Contributions

HI: study conception and design, analysis and interpretation of data, drafting of manuscript, critical revision, and final approval of the version to be published. CH: study design and data collection.

## Conflict of Interest Statement

The authors declare that the research was conducted in the absence of any commercial or financial relationships that could be construed as a potential conflict of interest.
